# Effect of CeO_2_ Addition on the Microstructure and Properties of Induction Heating Ni-WC-CeO_2_ Composite Coatings

**DOI:** 10.3390/ma18102175

**Published:** 2025-05-08

**Authors:** Lu Miao, Heqi Miao, Shangpeng Xie, Peibin Liu, Yanhui Li, Jihui Liu

**Affiliations:** 1 Institute of Applied Technology, University of Science & Technology Liaoning, Anshan 114051, China16641213505@163.com (P.L.); 15604207737@163.com (Y.L.); jihuiliu805930@163.com (J.L.); 2 Institute of Carbon Neutralization and Future Technology, China University of Petroleum (Beijing), Beijing 102249, China; 13238985826@163.com

**Keywords:** Ni-WC-CeO_2_ composite coating, CeO_2_ content, induction heating, microstructure, hardness, wear resistance

## Abstract

In this study, a BTG–15kW high-frequency induction heater was utilized to fabricate composite coatings of Ni-WC-CeO_2_ with varying CeO_2_ content on the surface of ASTM A36 steel substrates via induction cladding. The effects of CeO_2_ content on the phase composition, microstructure, elemental distribution, cross-sectional microhardness, surface hardness, Rockwell hardness, wear resistance, and wear scar morphology of the composite coatings were systematically examined using XRD, SEM, EDS, microhardness testers, Rockwell hardness testers, friction and wear testing machines, OM, and stylus profilers. The aim was to identify the optimal CeO_2_ content for enhancing coating performance. The results indicated that the incorporation of CeO_2_ promotes elemental inter-diffusion both within the coating and between the coating and the substrate, facilitates the dispersion of WC, and enhances the cross-sectional microhardness and wear resistance of the coating. However, excessive CeO_2_ content did not lead to further improvement, suggesting the presence of an optimal concentration. Among the compositions studied, the coating with 0.5% CeO_2_ exhibited minimal internal defects, pronounced elemental inter-diffusion, uniform WC, the highest cross-sectional microhardness and surface hardness, and the second-highest wear resistance, identifying this composition as the most effective for achieving superior coating performance.

## 1. Introduction

With the continuous development of modern industry, the performance demands placed on materials used in mechanical equipment have become increasingly stringent, Traditional single materials often fall short of meeting the comprehensive requirements for wear resistance, corrosion resistance, fatigue resistance, high-temperature oxidation resistance, and thermal shock resistance [1,2,3,4]. Therefore, material surface modification technology has gained prominence. This approach enhances the surface properties of materials without compromising the intrinsic characteristics of the substrate, thereby endowing the matrix materials with different excellent physical and chemical properties. Consequently, surface modification technologies have been widely applied in various fields such as industrial and agricultural production, marine vessels, aerospace, and others [5,6,7].

Induction heating is extensively employed in surface modification of metal materials due to its characteristics of rapid heating, less oxidation, and relatively simple equipment requirements. Coatings prepared using this method offer several benefits, such as high production efficiency, resistance to deformation, a high metallurgical bonding strength, dense structures, fewer defects, and a low cost [8]. As a result, they are extensively applied in aerospace, aviation, automobile engines, metallurgy, mechanical manufacturing, and marine engineering to enhance materials’ resistance to high-temperature oxidation, friction, and corrosion [9]. Although induction heating has a skin effect, using reasonable heating and heat-preservation power can effectively reduce the temperature difference between the surface and the core of the sample. Compared with laser cladding or argon tungsten-arc welding and surfacing, it reduces the composition difference between the front and rear weld overlaps, and welding and surface irregularities reduce a lot of work for subsequent coating-surface polishing, better metallurgical bonding and greater surface flatness by induction heating than thermal spraying.

Ni-based self-fluxing alloy powder exhibits good self-fluxing properties, wettability, abrasion resistance, corrosion resistance, and toughness. With a melting point temperature of 950~1050 °C, they are widely used in surface cladding and spraying modifications of steel substrates [10,11]. Yang Shangwu [12], Yu Kedong [13], Qu Zuoping [14], and others have employed various technologies, including laser heating, plasma cladding, thermal spraying, and induction heating, to deposit nickel-based self-fluxing alloys onto steel surfaces to investigate their abrasion resistance and corrosion resistance. Fossil Wei [15] and Zhao Ning [16], respectively, added Ti_3_SiC_2_ and nano La_2_O_3_ to the nickel-based alloy to evaluate their effect on the microstructure and overall performance of the resulting coatings.

CeO_2_ is a white rare earth oxide with a melting point of 2600 °C and excellent thermal stability. It can be used as an additive for coating materials to improve the mutual diffusion of elements between coating materials, refine the grain size, and improve the hardness, abrasion resistance, and corrosion resistance of the coating [17,18,19]. For instance, Xu Huanhuan [20] incorporated 1% CeO_2_ into a laser-clad nickel-based alloy, resulting in a coating with the finest microstructure, the highest hardness, and the best abrasion resistance. Similarly, Qin Nannan [21] added 1% CeO_2_ to a CoCrFeNiMo_0.2_ alloy coating, significantly improving its corrosion potential and resistance. Wu Zehua [22] achieved optimal hardness and a refined microstructure by introducing 0.3% CeO_2_ into a laser Ni-WC. Likewise, Yin Yan observed the finest grain structure and highest hardness in a laser-cladded Ni60A/TC4 composite layer with 0.3% CeO_2_ addition [23]. Based on the above research, this study employs ASTM A36 steel as the substrate and applies a Ni-Cr-Fe-WC-CeO_2_ composite coating using high-frequency induction heating. Five CeO_2_ concentrations, 0.0%, 0.25%, 0.5%, 0.75%, and 1.0% CeO_2_, are investigated to evaluate their effect on the coating’s microstructure and performance, with the aim of determining the optimal CeO_2_ content for enhanced coating properties.

## 2. Materials and Methods

### 2.1. Materials

ASTM A36 steel possesses good ductility, weldability, and a certain level of mechanical strength, and is widely used in various building and engineering structures [24]. We used normalized ASTM A36 steel with a 20 mm diameter and a height of 10 mm. The chemical composition of ASTM A36 steel is shown in Table 1.

The mixed powder for coatings is composed of Ni60A + 15% WC and 0%, 1%, 2%, 3%, and 4% CeO_2_ with at least 98% purity and an average particle size of 100 nm. After mechanical grinding with an agate mortar and infrared drying at 300 °C for 3 h, the 2.0 mm-high powder is preset on the bottom of a cylindrical ASTM A36 steel sample with a diameter of 20 mm. A BTG-15 kW high frequency induction heater is used to conduct induction cladding on five different component samples, the process parameters of induction heating are heating power of 4 kW, frequency of 30 kHz, heating time of 10s, insulation power of 2.1kW and air cooling to room temperature after 10 s. Finally, composite coatings with different amounts of dilute earth CeO_2_ added in 1.8 mm thickness are formed on the substrate surface. Five different component coatings are sampled and inlaid by wire cutting, polished by metallographic sandpaper, polished by grinding, cleaned by absolute ethyl alcohol ultrasonic waves, and dried for standby. Upon corrosion with a diluted aqua regia (with a volume ratio of concentrated hydrochloric acid to concentrated nitric acid of 1:3), the phase composition of the coating shall be detected with an X′Bowder XRD, and the microstructure of the coating section and the detection of energy-spectrum elements shall be observed with an Axis ZEISS optical microscope, a SIGMAHD SEM, and an EDS. Next, the microhardness of the sample section is tested with a Q10M micro-harness tester, with load of 0.2 kgf and holding time of 10 s. The joint between the coating and the matrix taken as the 0 position, the coating as positive direction. Points at 0.1 mm intervals from the matrix to the coating surface are taken as a coordinate position for testing. The average value of 5 points at the same coordinate position is taken. If there is a large deviation in the microhardness at a certain position, the next step is to remove the point and take another microhardness test to calculate the average value to represent the microhardness. A HR-150A Rockwell hardness tester is used to test the coating Rockwell hardness value, the test load is 150 kgf, measure the hardness of 5 points on the coating surface and calculate the average value. The chemical composition and properties of Ni60A and CeO_2_ are shown in Table 2 and Table 3, respectively.

### 2.2. Production-Processes Control

**Figure 1:**
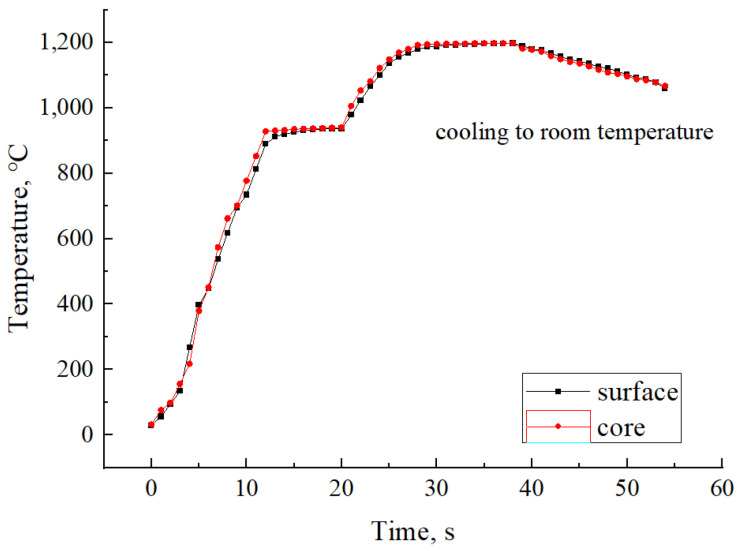
Rising Temperature curve.

The composite coating powders with different amounts of rare earth CeO_2_ were spread on the surface of the substrate ASTM A36 steel. Considering the characteristics of the skin effect of induction heating [25,26], graded high-frequency induction heating was adopted to better melt the cladding nickel-based alloy coating. The maximum heating temperature was 1180 °C, with a holding time of 10 s followed by natural cooling. A composite coating with a thickness of 1.8 mm was formed on the substrate surface. Due to the small amount of rare earth CeO_2_ added, the total mass of coating powder for one sample of each coating is 4.9800 g, calculated according to the volume and density of the Ni60A + 15% WC. According to the calculation results in Table 4, the mass of each powder is weighed with a balance with an accuracy of 0.0001 g. The mass of coating powder is shown in Table 4, and the induction heating temperature curve is shown in Figure 1.

## 3. Results and Discussion

### 3.1. Influence of CeO_2_ Addition on Coating Phase Composition

**Figure 2:**
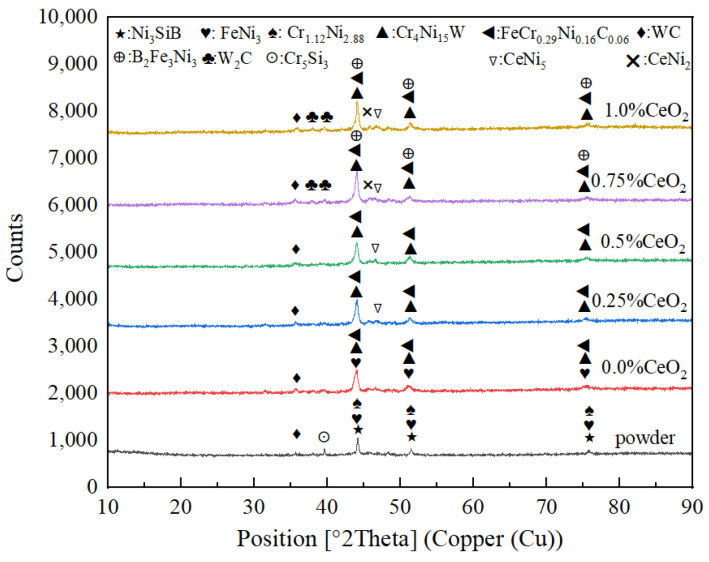
Result of XRD.

**Figure 3:**
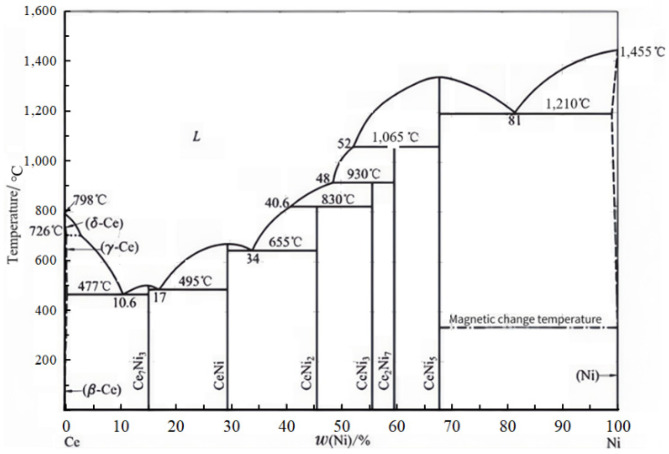
Ce-Ni binary phase diagram.

XRD phase-composition scanning of a Ni60A + 15% WC mixed powder and coatings with different CeO_2_ additions was performed by XRD, and the scanning results are shown in Figure 2. From Figure 2, it can be seen that the Ni60A+15% WC powder mainly consists of main-phase Ni_3_SiB (44.82°), FeNi_3_ (44.12°), Cr_1.12_Ni_2.88_ (44.28°), and secondary-phase Cr_5_Si_3_ (39.37°) WC (36.02°), and W4C (39.67°). After being made into a coating by induction heating, the WC and elements in Ni60A combine to form new main-phase Cr_4_Ni_15_W (43.85°), B_2_Fe_3_Ni_3_ (43.51°), and FeCr_0.29_Ni_0.16_C_0.06_ (43.58°). As the amount of CeO_2_ added to the coating increases, the WC in the coating decomposes into W_2_C phase [27]. Meanwhile, with the increase of CeO_2_ addition in the coating, CeNi_5_ (48.73°) and CeNi_2_ (46.61°) secondary phases are gradually generated. This is because the activation of the Ce element promotes the mutual diffusion between elements, so that more different elements generate diffusion and mutual solutions to form solid solution alloys, reducing the difference of different component phase elements. The Ce-Ni element will form a low-melting-point intermetallic compound in the metal melting pool, gradually moving to the grain boundary during the solidification process of the metal melting pool, and finally concentrating at the grain boundary to form CeNi_5_ and CeNi_2_ alloys. Ce-Ni phase diagrams [28,29] are shown in Figure 3.

### 3.2. Influence of CeO_2_ Addition on Macroscopic Morphology of Coating

**Figure 4:**
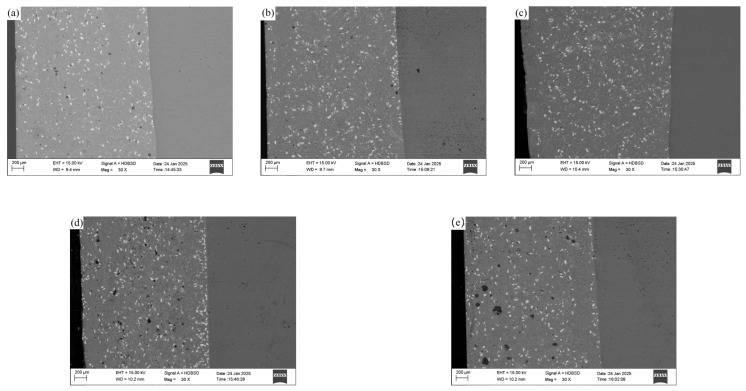
Macroscopic morphology of coatings. (**a**) 0.0% CeO_2_, (**b**) 0.25% CeO_2_, (**c**) 0.5% CeO_2_, (**d**) 0.75% CeO_2_, (**e**) 1.0% CeO_2_.

After cutting, the samples were embedded, ground, polished, and etched for metallographic examination. Due to the relatively large grain size of the iron-based coating, an optical microscope was used for observation. Figure 4a–e represents the macro-morphology of the coatings with rare earth CeO_2_ additions of 0.0%, 0.25%, 0.5%, 0.75%, and 1.0%, respectively, as shown in Figure 4. The left side of Figure 4 shows the composite coating, while the right side shows the ASTM A36 substrate. The white granular objects within the coating are the WC hard phase. From the five images, it is evident that the WC does not sink to the bottom of the coating due to its density of 15.63 g/cm^3^ being greater than that of the nickel-based self-fluxing alloy at 8.16 g/cm^3^ [30]. Instead, the WC is dispersed throughout the entire coating, with relatively more WC particles in the middle of the coating. This is because, in part, the magnetic susceptibility of the substrate Fe element is approximately 1.7 × 10^−4^ H/m, which is much greater than that of Ni at 0.6 × 10^−4^ H/m [31]. Therefore, during induction heating, the substrate is heated and warmed up more easily than the coating material. By heating the substrate material, the melting point of the coating material is reached, forming a metal melt pool on the surface of the substrate. Consequently, there is a thermal convection process from the bottom to the top within the metal melt pool, which helps the WC hard phase to disperse within the coating without sinking to the bottom due to its high density. On the other hand, an external medium-frequency magnetic field generates induced currents within the metal melt pool. The interaction between the induced current and the magnetic field produces electromagnetic forces that drive the stirring of the liquid metal within the melt pool, also helping to disperse the WC hard phase throughout the coating.

From Figure 4, it can also be observed that an appropriate addition of rare earth CeO_2_ can improve the internal quality of the coating. In Figure 4a, some porosity defects are visible in the cross-section of the coating. This is because there are numerous gaps between the pre-placed powder materials on the surface of the substrate, and these gaps are filled with air. Most of the gases escape from the metal melt pool during the heating process, but a very small amount of gas does not escape in time and ultimately forms porosity defects within the coating. After adding rare earth CeO_2_, which has good redox properties, into the coating, the oxygen element within the crystal can easily move or be missing, accompanied by changes in the valence state of Ce. Therefore, CeO_2_ can act as an oxygen buffer, effectively reducing the oxygen element that affects the surface tension of the coating, improving the fluidity of the coating, which is beneficial for the escape of gases from the metal melt pool and reduces the formation of porosity and inclusion defects [32]. Thus, the internal quality of the coating gradually improves with the increase in the addition of rare earth CeO_2_, as shown in Figure 4b,c. However, since CeO_2_ has a melting point of 2600 °C and good thermal stability, it can provide the metal melt pool with abundant oxygen vacancies, which can endow CeO_2_ with even better redox properties. Nevertheless, the energy that can be provided to the metal melt pool during the coating preparation process is limited. Therefore, when the addition of CeO_2_ is excessive, the internal quality of the coating counteractively becomes worse, and defects such as porosity and inclusions gradually increase instead, as shown in Figure 4d,e.

### 3.3. Effect of CeO_2_ Addition on Coating Bonding

**Figure 5:**
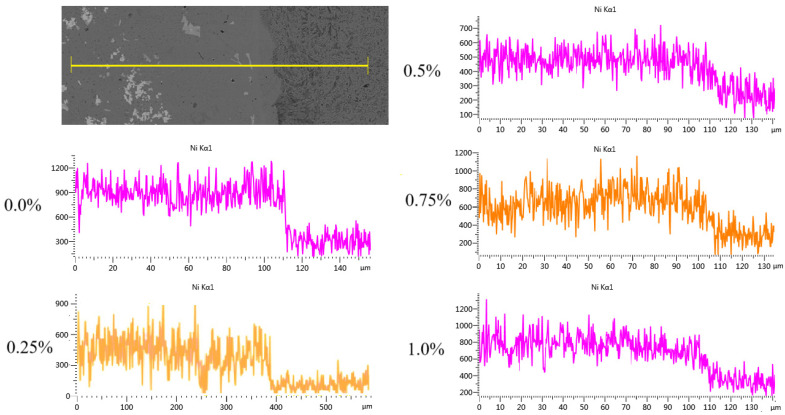
Joint line scanning of coatings.

The Ni element line scanning from the top of the coating to the inside of the matrix was carried out on each sample by using EDS, as shown in Figure 5, to study the diffusion of the coating elements through the matrix. It can be seen that the Ni element diffuses through the matrix in all samples. This is because the Ni atomic radius is 0.1241 nm, which is very similar to iron’s atomic radius 0.1243 nm [33]. It is an austenitic-forming element, and its solubility in γ-Fe is relatively high. Therefore, the Ni element can easily enter the matrix along the grain boundary and sub-grain boundary of the matrix, forming a replacement solid solution with a Fe element in the matrix, so as to form a good metallurgical bond between the coating and the matrix in the cladding process. It can be seen from the figure that the change of the diffusion-concentration gradient of Ni element in the CeO_2_ coating is slightly smaller than that in the non-CeO_2_ coating, indicating that CeO_2_ has the effect of promoting the diffusion of the coating through the matrix, because the arrangement of the electron layer of the Ce element is 4f15d16s2, and the special 4f layer vacancy is wrapped by 5d and 6s electrons, and it is very easy to lose 5d and 6s layer electrons [34], reducing the diffusion activation energy in the metal molten pool, effectively improving the mobility of the metal molten pool and the mutual diffusion of elements, and promoting the diffusion of the Ni element through the matrix.

**Figure 6:**
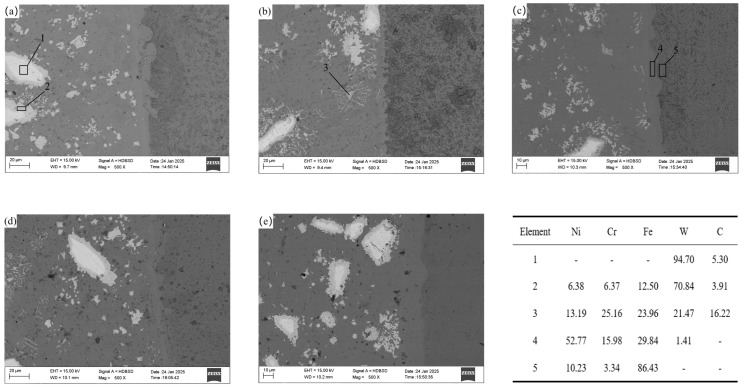
Microstructure and EDS of joints. (**a**) 0.0% CeO_2_, (**b**) 0.25% CeO_2_, (**c**) 0.5% CeO_2_, (**d**) 0.75% CeO_2_, (**e**) 1.0% CeO_2_, Wt.% in the table.

SEM was used to observe the microstructure at the joint of coating and carry out EDS scanning for different characteristic areas. The results are shown in Figure 6. The left side of all pictures is the coating structure, the white part is the WC hard phase particles, and the right side of the picture is the matrix of the ASTM A36. Since dilute aqua regia is used for corrosion, the matrix side metal has been completely corroded, and the structure cannot be seen clearly. It can be seen from all the pictures that the interface at the junction is not straight, which indicates that a mutual diffusion of elements has occurred between the matrix and the coating to form a metallurgical bond. EDS is used to analyze elements of the different morphologies of the WC and nickel-based alloys and matrices on both sides of the junction. Because the atomic weights of B, C, O, and Si are small, the energy spectrum results are relatively inaccurate. Except for the bulk WC, the above four kinds of non-metallic elements are removed. In Figure 6a, the hard phase EDS scanning in Area 1 of the WC shows that the internal WC has not been decomposed, and EDS scanning outside the core area of bulk WC particles in Area 2 also finds a large amount of Ni, Cr, and Fe elements. In Figure 6b,c, the WC particles in Figure 6a are relatively dispersed. In Figure 6b, a large amount of Ni, Cr, Fe, and other elements appear in the small block of WC in Zone 3 in the figure, and in Figure 6c, a large amount of W elements appear in Zone 4 in the figure, which indicates the mutual diffusion between the WC and nickel-base alloy. The WC in Figure 6b,c is detailed compared with Figure 6a, which indicates that adding rare earth CeO_2_ can promote the decomposition and diffusion of WC, because the difference between the atomic radius of Ce and Ni and Cr is far more than 15%, and the difference between the negativity of Ce and Ni is large [35]. The solid solubility in the Cr phase is relatively small. Under the energy effect of induction heating, the Ce atoms are solidly dissolved in the crystal lattice, grain boundary, or phase boundary with the increase of the amount of rare earth CeO_2_ added, so the system energy is reduced to the metastable state. From the thermodynamic point of view, the lattice distortion caused by the solid solution of the Ce atoms in the grain boundary zone is far less than that caused by the solid solution in the crystal. According to the principle of minimum energy, the Ce atom is easy to segregate near the WC hard phase and grain boundary [36]. It has the effect of purifying the interface of each phase in the coating, reducing the surface tension between the interfaces, improving the metal wettability and mobility between the interfaces, improving the density and uniformity of the coating alloy, effectively promoting the decomposition of the WC and reducing the size level of the WC and the CeO_2_. If an excessive amount is added, the capacity required for coating melting and diffusion increases continuously, the mobility of melt decreases, and the diffusion rate decreases during the solidification process of the molten pool. In Figure 6d,e, it can be seen that there is a large area of mutual diffusion between the bulk WC particles and the nickel-base alloy around the bulk WC particles. However, it can be seen in both figures that the bulk WC particles generate penetrating cracks inside the bulk WC particles because the surface of the WC particles in the metal molten pool becomes the crystal nucleus position of the nickel-base-alloy nucleation as a high melting point hard phase. The liquid nickel-base alloy gradually forms the nucleation and grows along the external surface, and the large tensile stress and the thermal stress generated due to temperature fluctuation are formed inside the bulk WC particles, exceeding the tensile limit, so the penetrating cracks are generated.

### 3.4. Effect of CeO_2_ Addition on Coating Microstructure

**Figure 7:**
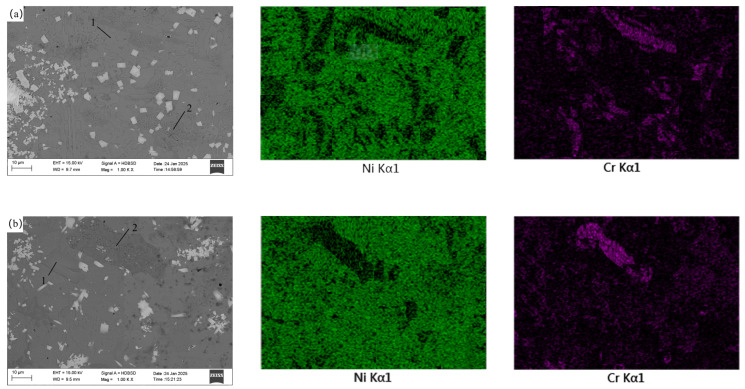

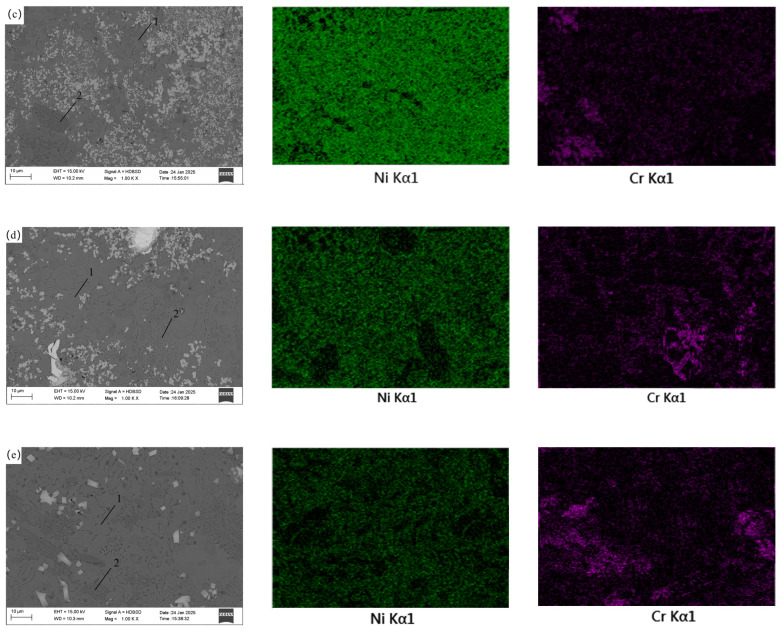
Microstructure in the middle of coatings. (**a**) 0.0% CeO_2_, (**b**) 0.25% CeO_2_, (**c**) 0.5% CeO_2_, (**d**) 0.75% CeO_2_, (**e**) 1.0% CeO_2_.

**Figure 8:**
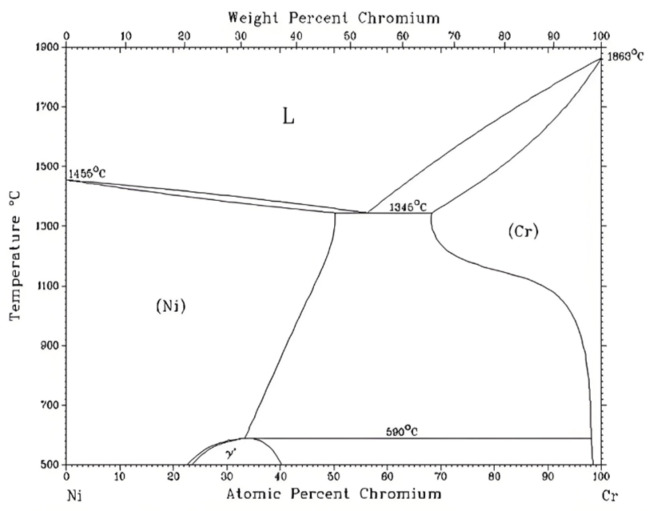
Ni-Cr binary phase diagram.

The microstructure and elemental distribution in the middle of the coating were detected using SEM and EDS, and the results are shown in Figure 7. Figure 7 also shows that adding an appropriate amount of CeO_2_ can reduce defects such as pores and inclusions within the coating, for reasons analyzed above. Compared to Figure 6, it can be seen that the WC particles in the middle of the coating are relatively fine. This is because WC with a smaller particle size, compared to larger particles, has a larger contact area with the nickel-based alloy. Under the action of electromagnetic force and thermal convection, they float to a relatively higher position in the metal melt pool, hence the WC particles in the middle of the coating are relatively fine. The microstructure in the middle of the coating can be roughly divided into blocky structure 1 and reticular structure 2. EDS was used to scan the elements of the two structures in coatings with different CeO_2_ additions, and energy spectrum analysis of the elements in coatings with different CeO_2_ additions was performed using EDS. Due to the small atomic weights of B, C, O, and Si, the energy spectrum results are relatively inaccurate. After removing the content of these four non-metallic elements, it was found that the blocky structure is a high-Ni phase, with an average Ni element mass content of about 75.2%, slightly higher than the Ni content in the nickel-based alloy powder, and the Cr element mass percentage is about 12.7%, slightly lower than the Cr content in the nickel-based alloy powder. The Fe element mass percentage is about 8.1%, slightly higher than the Ni content in the nickel-based alloy powder, and the remainder is the W element mass percentage, which corresponds to the Cr_4_Ni_15_W and B_2_Fe_3_Ni_3_ detected by XRD. The reticular structure is a high-Cr phase, with an average Cr element mass content of 55.4%, much higher than the Cr content in the nickel-based alloy powder; the Ni element mass content is 39.2%, lower than the Ni content in the nickel-based alloy powder; the Fe element mass percentage is 4.2%, slightly lower than the Fe content in the nickel-based alloy powder; and the remainder is W. The reason for the high Ni and high Cr phases can be seen from Figure 8 the Ni-Cr binary phase diagram. Ni-Cr binary alloys are infinitely miscible in the liquid phase, and in the solid phase [37], the Cr element mass percentage is within 56%, and the solidus line decreases with the increase of the Cr element mass percentage. Therefore, after cladding, during the cooling process of the metal melt pool, the solidification point temperature of the Ni-rich phase is higher than that of the Cr-rich phase. As the metal melt pool cools, the Cr-rich phase gradually moves outward from the blocky structure with the movement of the solid-liquid line, ultimately forming a Cr-rich reticular phase.

### 3.5. Effect of CeO_2_ Addition on Cross-Section Microhardness of Coating

**Figure 9:**
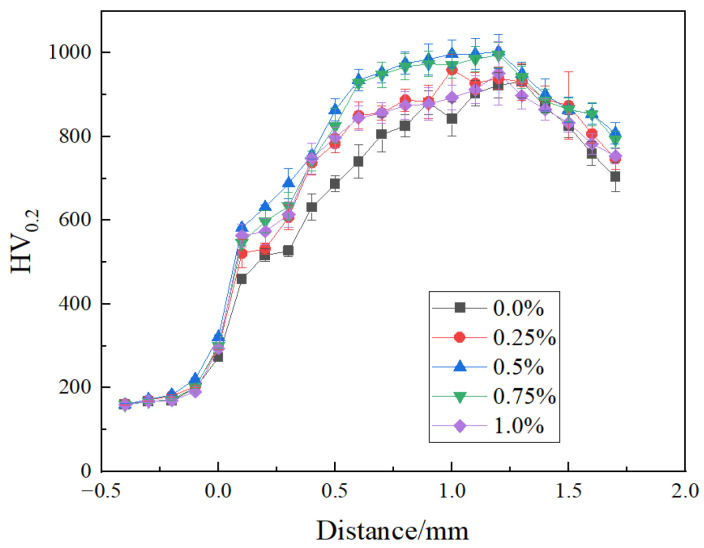
Coatings’ cross-section microhardness.

The microhardness test samples were grinded and polished on the surface, and the microhardness of the matrix, bonding area, and coating cross-section was detected using a microhardness tester. The test load was 0.2 kgf with a 10-s dwell time. The bonding area was taken as the “0” position, with the coating side being the “+” direction and the matrix side being the “−” direction. From the matrix toward the coating side, every 0.1 mm was taken as a step in distance, and the microhardness of three different positions at this step distance was measured. The average value of these three measurements was taken as the microhardness at that step distance. If a measurement point encountered a WC particle during measurement, or if the microhardness was significantly different from other points at the same step distance, that point was removed, and another microhardness value was taken, and then the average was recalculated. Although the coating thickness was 1.8 mm, due to the presence of many defects such as pores and slag on the coating surface, the hardness-measurement position coordinates on the coating side were only measured up to 1.7 mm away from the matrix. The microhardness test results of the composite coating cross-section are shown in Figure 9.

From Figure 9, it can be seen that the microhardness of the nickel-based coating side of all samples is much higher than that of the matrix side. On the one hand, this is because the nickel-based alloy contains a large amount of alloying elements such as Cr, W, and Fe, which can form solid solutions with Ni, significantly improving the hardness of the nickel-based alloy through solid solution strengthening mechanisms [38]. On the other hand, during the cladding and crystallization process of the nickel-based alloy, some alloying elements precipitate from the solid solution, forming fine secondary phase particles that further hinder dislocation movement and increase the hardness of the material. By observing the microhardness curves of all samples, it can be found that the microhardness on the coating side first increases and then decreases, i.e., the microhardness curve shows a “mountain” shape distribution. This is because, in the induction heating cladding process, under the action of thermal convection and electromagnetic stirring, the relatively large volume of WC particles is relatively close to the coating joint, while the relatively small volume of WC particles is relatively close to the coating top, while relatively small WC particles are closer to the top of the coating. Fine WC particles can not only play a better role in dispersion strengthening, but also more easily diffuse through the nickel-based alloy, playing a role in solid-solution strengthening, thus causing the coating microhardness to first increase. During the coating induction-heating cladding process, bubbles and low-density slag generated in the metal melt pool float up to the top of the coating, resulting in more crystal defects at the coating surface and subsurface, thus causing a relative decrease in the microhardness of the coating surface and subsurface.

From Figure 9, it can also be seen that the microhardness of the matrix with added rare earth CeO_2_ is higher than that of the matrix without addition. The reason is the addition of rare earth CeO_2_ promotes the diffusion of elements such as Ni and Cr into the matrix, forming solid solution alloys with Fe elements at that location, resulting in solid-solution strengthening and an increase in matrix microhardness. Moreover, by comparing the curves, it can be seen that under the induction-heating conditions of this experiment, the depth of diffusion of coating elements into the matrix is approximately 0.4 mm. The microhardness inside the coating without added CeO_2_ is lower than that of the coating with added CeO_2_. When the CeO_2_ addition amount is 0.5%, the microhardness inside the coating is the highest, and the hardness fluctuation is relatively small. This is because the coating with a 0.5% addition has the fewest defects inside, the WC particles inside the crystal are fine and dispersed, and there is a significant element inter-diffusion phenomenon between the WC particles and the nickel-based alloy, which improves the coating microhardness. When the CeO_2_ addition amount exceeds 0.5%, the dispersion degree of the WC particles decreases, and the number of defects inside the coating increases, which leads to a corresponding decrease in the hardness inside the coating and a relatively larger fluctuation in hardness.

### 3.6. Effect of CeO_2_ Addition on Wear Resistance of Coating

**Figure 10:**
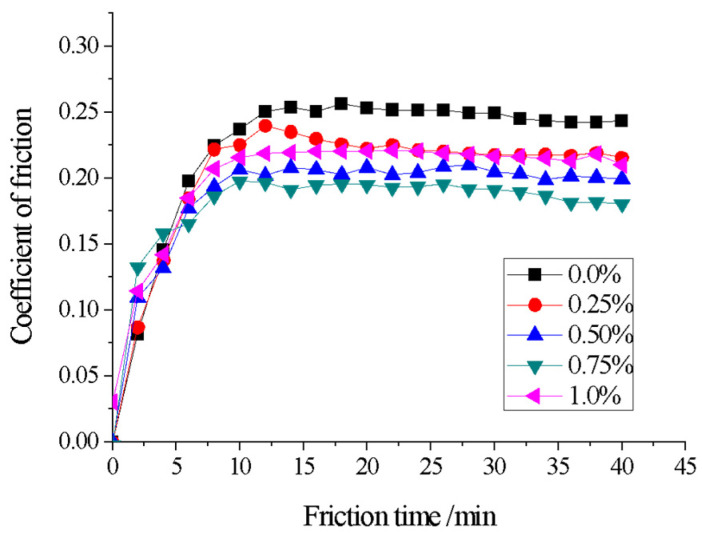
Friction coefficient curve.

After grinding off defects such as slag and pores on the surface of coatings with different CeO_2_ additions, fine grinding and polishing were performed on the coating layers. The wear resistance of coating samples with different CeO_2_ additions was tested using MS-T3001 friction and a wear-testing machine. The counter-body used was ZrO_2_ with a diameter of 3 mm, the test load was 5 N, and the wear time was 40 min. The friction coefficient wear-time curve was plotted based on the data collected by the friction and wear-testing machine, and the results are shown in Figure 10. From Figure 10, it can be seen that all coating samples undergo a running-in wear stage and a stable wear stage. In the initial operation of the new friction pair, due to the large surface roughness value of the mating surface, the actual contact area is small, the number of contact points is few, and the area of most contact points is large, resulting in severe adhesion at the contact points, and therefore, the wear rate is high. However, as the running-in progresses, the surface micro-peaks are gradually ground away, the surface roughness value decreases, the actual contact area increases, the number of contact points increases, the wear rate decreases, and it gradually enters a stable wear stage where the wear rate remains basically unchanged [39,40].

**Figure 11:**
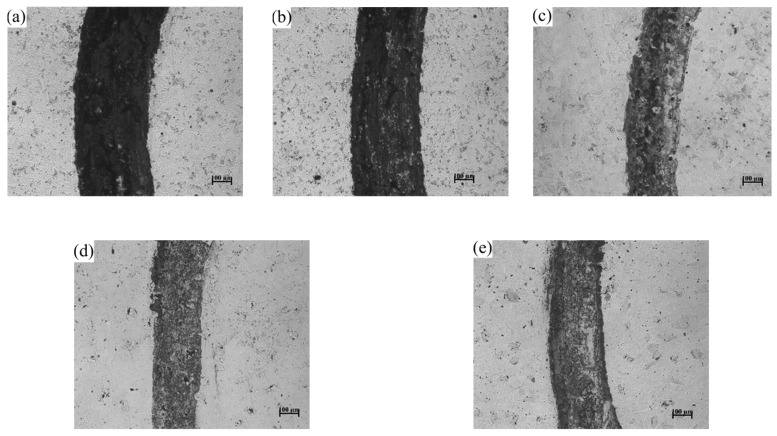
Coatings’ abrasion mark. (**a**) 0.0% CeO_2_, (**b**) 0.25% CeO_2_, (**c**) 0.5% CeO_2_, (**d**) 0.75% CeO_2_, (**e**) 1.0% CeO_2_, Wt.% in the table.

As can also be seen from Figure 10, the surface friction coefficient of the coatings with CeO_2_ is lower than that of the uncoated surfaces, indicating an improvement in the friction resistance of the coatings. The surface friction coefficient of the coatings also exhibits a trend of first decreasing and then increasing with the increase of CeO_2_ addition. When the CeO_2_ addition is 0.75%, the friction coefficient is the lowest, and the friction resistance of the coating is the highest. This can also be observed in the wear scar morphology shown in Figure 11. The wear scar width follows the order (a) 0.387 mm > (b) 0.367 mm > (c) 0.24 mm > (d) 0.22 mm < (e) 0.267 mm. The wear scar morphology of the coatings was drawn using the Alpha-Step D600 Stylus Profiler, and the wear rates of the various coatings were calculated using empirical formulas, as shown in Table 5. The calculation formula is shown in Formula (1), where: *B*—width of wear mark/mm; *h*—depth of wear mark/mm; *F*—friction load/N; *n*—rotating speed of testing machine/r·min^−1^; *t*—Experimental time/min.

(1) $$\delta =\frac{\Delta V}{FL}=\frac{Bh}{Fnt}$$

**Figure 12:**
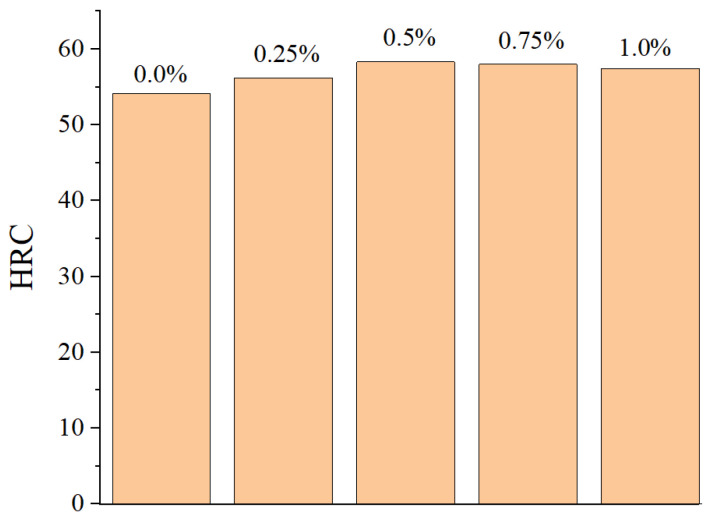
Rockwell hardness.

From Table 5 and Figure 10, it can be observed that the addition of an appropriate amount of CeO_2_ can reduce the friction coefficient of the coating surface and decrease the wear rate. This is influenced by both the coating’s cross-sectional microhardness and the surface Rockwell hardness. The greater the coating’s microhardness, the more wear resistant it becomes. The surface Rockwell hardness of the coating is shown in Figure 12. On the other hand, as can be seen in Figure 11c–e, adding an appropriate amount of CeO_2_ to the coating can effectively promote the diffusion of WC hard particles through the nickel-based alloy, forming a stable metallurgical bond between the two components. During the friction and wear process, when the friction pair encounters the hard phase WC particles, it cannot form an adhesive layer, effectively preventing adhesive wear of the friction pair [41]. At the same time, the WC hard phase particles can also reduce the formation of micro-cracks on the coating surface under alternating loads, further preventing the occurrence of fatigue wear, resulting in a significant reduction in coating wear.

## 4. Conclusions

(1) The addition of rare earth CeO_2_ to nickel-based composite coatings promotes the diffusion of alloy elements such as Cr and Ni into the substrate, resulting in a good metallurgical bond between the coating and the substrate.

(2) The addition of CeO_2_ facilitates the decomposition of WC into W_2_C in the coating and generates low-melting-point alloys of CeNi_5_ and CeNi_2_ in the coating as the addition of CeO_2_ increases.

(3) An appropriate amount of CeO_2_ addition enhances the fluidity of the coating, reduces internal pores and inclusions, promotes the fine and dispersed distribution of WC particles, and improves the microhardness of the coating cross-section and the Rockwell hardness of the coating surface.

(4) An appropriate amount of CeO_2_ addition can reduce the surface friction coefficient of the coating and enhance the surface wear resistance of the coating.

(5) The coating with 0.5% CeO_2_ added has the best internal quality and inter-diffusion of elements between the coating and the substrate, the highest microhardness of the coating cross-section, the highest Rockwell hardness of the surface, and the second-highest wear resistance, making it the optimal addition amount for this composite coating.

## Figures and Tables

**Table 1:** Chemical composition of ASTM A36 steel.

Element	C	Mn	Si	P	S	Cu	Fe
Wt./%	≤0.25	0.80~1.20	≤0.40	≤0.04	≤0.05	≥0.20	Bal.

**Table 2:** Chemical composition of Ni60A self-fusing alloy powder.

Substances and Properties	C	Cr	Si	B	Fe	Ni	Mesh
Wt./%	0.5~1.1	15~20	3.5~5.5	3.0~4.5	≤5.0	Bal.	−150~+320

**Table 3:** Chemical composition and properties of CeO_2_ powder.

Substances and Properties	Cu	Fe	Nitric Acid Insoluble	Other Rare Earths	Burn Weightless	Purity
Wt./%	<0.002	<0.002	<0.03	<0.5	<0.2	≥99

**Table 4:** Powder Mass of Each Coating.

Powder Material	0.0% CeO_2_	0.25% CeO_2_	0.5% CeO_2_	0.75% CeO_2_	1.0% CeO_2_
Ni60A + 15% WC, Wt./g	4.9800	4.9675	4.9551	4.9426	4.9302
CeO_2_, Wt./g	0	0.0125	0.0249	0.0374	0.0498

**Table 5:** Coating wear rate.

	(a)	(b)	(c)	(d)	(e)
Rate of wear/mm^3^·N^−1^·m^−1^	2.2	1.9	1.6	1.5	1.8

## Data Availability

The original contributions presented in this study are included in the article. Further inquiries can be directed to the corresponding author(s).
